# Preemptive Low-Dose Norepinephrine Infusion for Reducing Hemodynamic Instability During Craniotomy for Brain Tumor Resection Under Propofol–Remifentanil Total Intravenous Anesthesia: A Randomized Controlled Trial

**DOI:** 10.3390/jcm15135046

**Published:** 2026-06-29

**Authors:** Kyeong Tae Min, Seung Ho Choi, Hyun Joo Kim, Bahn Lee, Seungyeon Lee, Hye Jin Kim

**Affiliations:** Department of Anesthesiology and Pain Medicine, Anesthesia and Pain Research Institute, Yonsei University College of Medicine, Seoul 03722, Republic of Korea; ktmin501@yuhs.ac (K.T.M.); csho99@yuhs.ac (S.H.C.); jjollong@yuhs.ac (H.J.K.); sylee12@yuhs.ac (S.L.)

**Keywords:** norepinephrine, hypotension, hypertension, propofol, neurosurgery

## Abstract

**Background:** Previous trials of prophylactic norepinephrine have compared it with volume loading or non-norepinephrine vasopressors. Thus, it remains unclear whether a preemptive norepinephrine strategy provides incremental benefit over conventional management permitting reactive norepinephrine use. We evaluated whether preemptive low-dose norepinephrine infusion reduces hemodynamic instability during craniotomy for brain tumor resection under propofol–remifentanil total intravenous anesthesia (TIVA). **Methods:** Adult patients undergoing craniotomy for brain tumor resection under propofol–remifentanil TIVA were randomized to preemptive continuous infusion of norepinephrine (CINE; started at 0.02 µg/kg/min and titrated to remain below 0.05 µg/kg/min) or conventional management, in which norepinephrine was administered at the anesthesiologist’s discretion in response to hypotension. The primary endpoint was moderate or severe hemodynamic instability, defined as mean arterial pressure outside 80–120% and 70–130% of baseline, respectively. Secondary endpoints included rescue medication use, postoperative complications, and safety. **Results:** Compared with conventional management, the CINE group showed less moderate hemodynamic instability, both in the number of episodes per patient (median [interquartile range]: 6 [3–11] vs. 9 [5–13], *p* = 0.045) and in the proportion of anesthesia time affected (7.4% [3.6–12.4] vs. 12.2% [6.8–21.4], *p* = 0.017), but not less severe instability. Rescue medication was required less frequently in the CINE group (16.7% vs. 100%, *p* < 0.001). Complication rates were similar between the groups, and no adverse drug reactions occurred. **Conclusions:** Preemptive low-dose norepinephrine infusion reduced moderate hemodynamic instability during propofol–remifentanil TIVA for brain tumor resection, even against a control group receiving conventional management with reactive norepinephrine use, suggesting potential hemodynamic benefit that warrants confirmation in larger trials.

## 1. Introduction

Intraoperative hypotension (IOH) is frequently encountered during general anesthesia, with reported incidence varying widely according to the definition applied [1]. When IOH exceeds a certain threshold in severity or duration, organ perfusion is compromised, leading to increased morbidity [2].

This issue is particularly relevant during craniotomy for brain tumor resection. In these patients, adequate cerebral perfusion must be maintained while avoiding excessive hypertension that may worsen brain swelling or increase bleeding risk. Brain tumors are frequently associated with blood–brain barrier disruption and vasogenic edema [3], and surgical manipulation may further alter local cerebral physiology. Accordingly, tight hemodynamic control is a key anesthetic goal during brain tumor surgery.

Propofol–remifentanil total intravenous anesthesia (TIVA) is commonly used in brain tumor surgery because it facilitates intraoperative neuromonitoring and brain relaxation [4]. However, this anesthetic regimen is frequently accompanied by hypotension, as propofol and remifentanil can reduce systemic vascular tone and myocardial contractility, at least in part through suppression of sympathetic activity and circulating norepinephrine release [5]. In this context, low-dose norepinephrine is physiologically appealing because it may counteract anesthesia-induced vasodilation and support effective circulating volume while the vasodilatory effects of anesthesia persist [6,7]. This rationale is supported by experimental and clinical physiological data: deep propofol sedation in healthy volunteers reduced blood pressure by approximately 18% together with plasma norepinephrine levels [8], whereas a norepinephrine infusion of approximately 0.05 µg/kg/min was shown to maintain a mean arterial pressure of 65 mmHg under propofol–remifentanil anesthesia [9].

Previous studies have demonstrated that prophylactic norepinephrine infusion reduces intraoperative hypotension across several surgical settings [10,11,12]. However, these studies compared norepinephrine against volume loading or non-norepinephrine vasopressors, and thus do not address the clinically relevant question of whether a preemptive strategy confers incremental benefit over reactive administration of norepinephrine. This comparison has growing relevance given the increasing recognition of norepinephrine as a physiologically preferable vasopressor for anesthesia-induced hypotension [5], yet remains unanswered in neurosurgical patients undergoing propofol–remifentanil TIVA.

We therefore conducted a randomized controlled trial to evaluate whether preemptive continuous low-dose norepinephrine infusion, incorporated into a standardized propofol–remifentanil TIVA protocol, reduces the burden of intraoperative hemodynamic instability during craniotomy for brain tumor resection compared with conventional management permitting reactive norepinephrine use.

## 2. Materials and Methods

### 2.1. Ethics

This randomized controlled trial was conducted at Severance Hospital, Yonsei University Health System, Seoul, Korea, in accordance with the Declaration of Helsinki. The study was approved by the Institutional Review Board of Severance Hospital, Yonsei University Health System (IRB no. 4-2022-1619; approved in 15 February 2023) and was registered at ClinicalTrials.gov (NCT05814601) on 18 April 2023, before patient enrollment (on 20 June 2023). Written informed consent was obtained from all patients. This manuscript adheres to the CONSORT guidelines.

### 2.2. Study Design and Patients

Patients were enrolled between June 2023 and March 2024. Eligible participants were patients aged 20–65 years scheduled for elective craniotomy for primary brain tumor resection. Exclusion criteria were as follows: (1) cognitive impairment; (2) pregnancy or lactation; (3) inability to provide written informed consent because of illiteracy or a language barrier; (4) emergency surgery; (5) arrhythmia; (6) congestive heart failure (New York Heart Association class ≥ 3); (7) uncontrolled hypertension (baseline blood pressure > 140/90 mmHg); (8) renal dysfunction (estimated glomerular filtration rate < 30 mL/min/1.73 m^2^); and (9) severe respiratory disease (pneumonia, chronic obstructive pulmonary disease, or asthma requiring treatment).

### 2.3. Randomized Allocation

An anesthesiologist generated the allocation sequence using a random number generator in Microsoft Excel 2016 (Microsoft Corp., Redmond, WA, USA). Fixed blocks of four were used in a 1:1 ratio, except for the final block, which contained two patients. Patients were assigned to either the continuous infusion of norepinephrine (CINE) group or the conventional (CONV) group.

### 2.4. Anesthesia Protocol by Group

Upon arrival in the operating room, patients were monitored using a three-lead electrocardiogram, noninvasive blood pressure monitor, and pulse oximeter. After establishing monitoring, glycopyrrolate (4 µg/kg, up to a maximum of 0.2 mg) was administered intravenously. Patients received 100% oxygen via a mask for at least 3 min.

For the anesthesia protocol of the CONV group, anesthesia induction and maintenance were performed using TIVA with propofol (Fresofol 2% MCT; Fresenius Kabi Austria GmbH, Graz, Austria) and remifentanil (Ultian; Hanlim Pharm. Co., Ltd., Seoul, Republic of Korea) via a target-controlled infusion system. The target effect-site concentration of propofol was set at 3–4 µg/mL, whereas that of remifentanil was maintained at 3–4 ng/mL, with adjustments at the discretion of the attending anesthesiologist. Upon loss of consciousness, rocuronium (0.6–1.0 mg/kg; Rocumeron; Ilsung Pharmaceuticals Co., Ltd., Seoul, Republic of Korea) was administered, followed by mask ventilation with 100% oxygen. “Adequate neuromuscular blockade was confirmed by the absence of response to train-of-four stimulation of the ulnar nerve at the adductor pollicis muscle, assessed using a peripheral nerve stimulator (Innervator 252; Fisher & Paykel Healthcare, Auckland, New Zealand). The trachea was intubated, and an arterial catheter was inserted into the radial artery for real-time arterial pressure monitoring. Noninvasive blood pressure was measured at 2 min intervals until arterial cannulation. Initial ventilator settings were established with a tidal volume of 6 mL/kg (ideal body weight), respiratory rate of 14 breaths/min, and positive end-expiratory pressure of 5 cmH_2_O. Throughout anesthesia, the bispectral index was targeted at 40–60.

In the CINE group, norepinephrine (stock concentration, 5 µg/mL) was co-administered in addition to the same TIVA protocol used in the CONV group. The preemptive norepinephrine infusion was initiated at 0.02 µg/kg/min and titrated to remain below 0.05 µg/kg/min while anesthetic depth and mean blood pressure (MBP) were kept within their target ranges. The infusion was administered through a peripheral venous catheter (20-gauge or larger, placed at least 2 inches above the wrist) or, when available, a central venous catheter. Norepinephrine administration began once the target effect-site concentration of propofol had been achieved and was tapered off following the discontinuation of anesthetic agents and completion of surgery. The peripheral infusion site was inspected hourly for local adverse events, such as erythema, blanching, or swelling.

The lowest MBP measured on the ward on the day before surgery was used as the baseline value. Intraoperative hemodynamic management aimed to maintain MBP within 80–120% of baseline. A decrease in MBP to below 80% of baseline was regarded as IOH and was managed with fluid administration or vasopressor treatment at the discretion of the attending anesthesiologist, guided by dynamic parameters such as pulse pressure variation (PPV) and stroke volume variation (SVV). In the CINE group, rescue vasopressor use was defined as norepinephrine administration exceeding 0.05 µg/kg/min or administration of a vasopressor other than norepinephrine, whereas in the CONV group it was defined as any vasopressor administration. If MBP exceeded 120% of baseline, the concentrations of propofol and remifentanil were increased, and antihypertensive agents were administered as rescue medication at the discretion of the attending anesthesiologist, in accordance with standard practice. Plasma lactate levels were measured immediately after anesthesia induction, intraoperatively, and at the end of surgery. After surgery, patients were transferred to the neurocritical care unit (NCU) while still intubated, and blood pressure, electrocardiography, and oxygen saturation were continuously monitored. Dropout criteria were as follows: (1) withdrawal of consent; (2) a change in the surgical plan; (3) more than two consecutive missing MBP measurements, and (4) intraoperative transfusion of more than two units of packed red blood cells to exclude hemodynamic instability primarily attributable to surgical causes.

Attending anesthesiologists were aware of group allocation to ensure patient safety. However, for the analysis, data were provided to the analyst with the treatment groups coded as “A” and “B”, thereby maintaining blinding at the analysis stage.

### 2.5. Study Endpoints

The primary endpoint was the occurrence of moderate or severe hemodynamic instability, defined as MBP deviations beyond 80–120% and 70–130% of baseline MBP, respectively. Because at least one episode of moderate hemodynamic instability occurred in all patients, the primary comparison focused on the number of episodes and the proportion of anesthesia time spent outside the target MBP range, rather than incidence alone. Secondary endpoints included the need for rescue medication for hemodynamic instability, postoperative complications, and local adverse events related to preemptive norepinephrine infusion, such as erythema, blanching, or swelling of the skin. Postoperative complications included pleural effusion, atelectasis, respiratory infection, acute kidney injury, myocardial infarction, new-onset cardiac arrhythmia, postoperative hemorrhage, stroke, new neurological deficit, unexpected readmission to the NCU or operating room during the study period, and 30-day mortality after surgery (Table S1 in Online Resource 1) [13]. Postoperative NCU stay and total hospital stay were also recorded.

### 2.6. Sample Size Calculation

Given the lack of prior studies using a definition of hemodynamic instability identical to ours, we referred to a study in a comparable setting—patients undergoing TIVA with propofol for elective intracranial surgery—which reported a 62.5% incidence of hypotensive episodes (defined as MBP < 60 mmHg for > 1 min unresponsive to fluid bolus) [14].

Similarly, because at the time of study design in 2023, no RCT had directly reported the effect of prophylactic norepinephrine on hypotension incidence in patients undergoing general anesthesia, the expected treatment effect was derived from the best available indirect evidence: prophylactic norepinephrine infusion during spinal anesthesia for cesarean delivery, which demonstrated a relative risk reduction of approximately 74% in hypotension incidence [15]. To account for the differences in surgical setting and patient population, we applied a more conservative estimate of 60% relative risk reduction, yielding an expected reduction in hemodynamic instability from 62.5% to 25%. Based on this assumption, a sample size of 31 patients per group was calculated using a two-tailed significance level of 5% (α = 0.05) and 80% power (β = 0.20). Accounting for a 10% dropout rate, a total of 70 patients (35 per group) were required.

### 2.7. Data Curation and Statistical Analysis

Intraoperative blood pressure and heart rate were continuously monitored and recorded in electronic medical records (EMRs). Hemodynamic data were extracted later for analysis at 5 min intervals from the EMR. Postoperative complications were investigated based on the EMR documented by the neurosurgeons and nursing staff, as well as the radiographic and laboratory data.

Hemodynamic data were screened for missing values and implausible outliers. When up to two consecutive MBP measurements were missing, values were imputed using the average of the preceding and following MBP readings. For data deemed inconsistent with clinical context (e.g., measurement errors due to transducer misplacement), anesthesia records were jointly reviewed by the attending anesthesiologist and data collector. Confirmed erroneous values were treated as missing.

An absolute standardized difference of >0.494 indicated an imbalance in baseline characteristics [16,17]. Continuous variables were analyzed using the independent *t*-test or Mann–Whitney U test after assessing normality with the Shapiro–Wilk test. Normally and non-normally distributed continuous data are presented as the mean ± standard deviation and median (interquartile range [IQR]), respectively. The estimated median difference and 95% confidence interval (CI) were computed using bootstrapping, which involved random resampling with replacement with 10,000 replicates. Categorical variables were analyzed using the chi-square or Fisher’s exact test and are presented as numbers (%). For categorical variables with more than two subcategories, a chi-square test with Monte Carlo simulation was performed using 10,000 simulations. Relative risk (RR) and 95% CI were calculated using the epiR package in R, specifically employing the epi.2by2 function with the “cohort.count” method. The risk ratio was log-transformed to obtain the standard error, which was then used to construct the CIs. These intervals were subsequently back-transformed to the original scale for interpretability. Changes in plasma lactate levels were analyzed using a linear mixed-effects model. A random intercept for subjects was included to account for repeated measures within individuals. The group and time point were included as fixed effects in the model, which was fitted using the nlme package in R. Baseline plasma lactate levels measured at anesthetic induction were included in the model as a covariate, thereby adjusting for baseline differences in lactate concentration. Estimated marginal means with 95% confidence intervals, adjusted for baseline values, were obtained using the emmeans package, and Bonferroni-adjusted pairwise comparisons between groups were performed at each time point.

All analyses followed an intention-to-treat approach. Statistical analyses were performed using the Statistical Package for the Social Sciences version 27 (IBM Corp., Armonk, NY, USA) and R version 4.4.1 (The R Foundation for Statistical Computing, Vienna, Austria). A *p*-value < 0.05 was considered statistically significant.

During the preparation of this manuscript, the authors used ChatGPT (GPT-5.5; OpenAI, San Francisco, CA, USA) and Claude (Sonnet 4.6; Anthropic, San Francisco, CA, USA) for language editing and improving readability, and for assisting in the preparation of the graphical abstract. The authors have reviewed and edited the output and take full responsibility for the content of this publication.

## 3. Results

**Figure 1:**
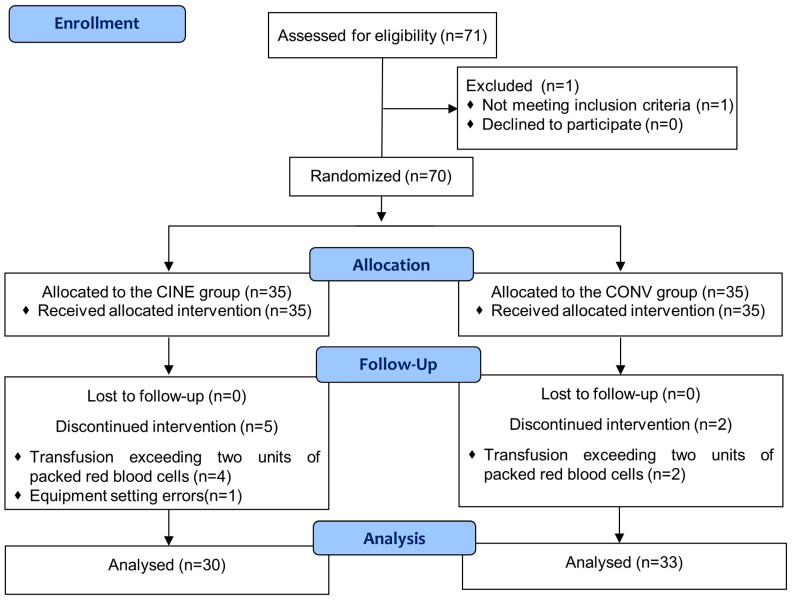
Flow diagram of patient enrollment. CINE, continuous infusion of norepinephrine; CONV, conventional.

Of the 71 patients assessed for eligibility, 70 were enrolled in the study. Among these, seven patients were excluded: six who received more than two units of packed red blood cells intraoperatively and one who received an incorrect dose of propofol due to an equipment setting error. Ultimately, 63 patients completed the study: 30 in the CINE group and 33 in the CONV group (Figure 1).

Demographic and operative characteristics are presented in Table 1 and Table 2, respectively. Although tumor location differed between the groups, the closely related surgical position did not (*p* = 0.294), and other baseline characteristics—including age, sex, tumor type, duration of anesthesia, and estimated blood loss—were comparable. Baseline MBP was 89 ± 7 mmHg in the CINE group and 88 ± 9 mmHg in the CONV group, with no significant between-group difference.

Outcome data on intraoperative hemodynamic instability and rescue medication use are shown in Table 3. Moderate hemodynamic instability occurred in all patients, but the CINE group experienced fewer episodes per patient (median: 6 [IQR: 3–11] vs. 9 [5–13]; *p* = 0.045) and a lower proportion of affected anesthesia time (7.4% [3.6–12.4] vs. 12.2% [6.8–21.4]; *p* = 0.017). The incidence of severe hemodynamic instability was similar between groups, with no significant difference in the number of affected patients (63.3% vs. 72.7%; *p* = 0.424), episodes per patient (1 [0–2] vs. 2 [0–3]; *p* = 0.285), or the proportion of affected anesthesia time (1.2% [0.0–2.2] vs. 1.8% [0.0–3.8]; *p* = 0.127). The need for rescue medication was significantly lower in the CINE group (5 patients [16.7%] vs. 33 patients [100%]; RR: 0.17, 95% CI: 0.07–0.37; *p* < 0.001). Neither group required rescue medications for hypertensive events.

After adjustment for baseline lactate levels, plasma lactate levels over time did not differ significantly between the CINE and CONV groups (*p* for group-by-time interaction = 0.203; Bonferroni-adjusted pairwise comparisons at each time point are presented in Table 4). No significant between-group differences were observed in postoperative morbidity or 30-day mortality (Table S2 in Online Resource 1). No local adverse events, including erythema, blanching, or swelling, were noted at the peripheral infusion sites.

## 4. Discussion

In this randomized controlled trial, preemptive low-dose norepinephrine infusion reduced the frequency and cumulative time burden of moderate hemodynamic instability during craniotomy for brain tumor resection under propofol–remifentanil TIVA, even when compared against a control group permitting reactive norepinephrine administration. To our knowledge, this is the first RCT to compare preemptive versus reactive vasopressor management in a neurosurgical TIVA population. The benefit was limited to moderate instability and is best interpreted as improved intraoperative hemodynamic control—a process-of-care measure—rather than a demonstrated effect on clinical outcomes, as no significant differences were observed in severe instability or postoperative morbidity.

The reduction in moderate hemodynamic instability has potential clinical significance. The absolute reduction in affected anesthesia time—from 12.2% in the CONV group to 7.4% in the CINE group, corresponding to approximately 18 min over a median anesthetic duration of more than 6 h—represents meaningful improvement in intraoperative hemodynamic control, given that intraoperative hypotension defined as a reduction of approximately 20% below baseline is independently associated with postoperative organ injury in a graded, time-dependent manner [2,18,19]. While the absence of detectable differences in postoperative complications may partly reflect the limited statistical power of this study, the clinical implications of improved hemodynamic control remain to be established in adequately powered trials.

Several factors may explain why the benefit was confined to moderate, but not severe, hemodynamic instability. The conservative dose range used in our protocol, combined with the mismatch between anesthetic depth and varying surgical stimulation, likely contributed to this pattern. During craniotomy, surgical stimulation can vary substantially, ranging from high-intensity phases such as head pinning and bone cutting to lower-intensity phases such as brain tissue manipulation. Despite these fluctuations, relatively deep anesthesia may be required to prevent patient movement, particularly when neuromuscular blockade is minimized or avoided to facilitate motor-related neurophysiological monitoring. Under these conditions, the physiologic low-dose norepinephrine infusion used in our study may have been sufficient to attenuate moderate anesthesia-induced vasodilation and hypotension, but insufficient to prevent larger blood pressure deviations during periods of relatively deep anesthesia, abrupt changes in surgical stimulation, or other procedure-related hemodynamic perturbations.

Several studies have evaluated prophylactic norepinephrine in surgical patients [10,11,12], but important differences distinguish the present trial from previous work. First, our study focused on a homogeneous neurosurgical population undergoing craniotomy for brain tumor resection under propofol–remifentanil TIVA—a setting with uniquely constrained hemodynamic targets in which both hypotension and hypertension carry distinct cerebrovascular risks—rather than on a broader mixed surgical population. Second, whereas some previous studies used broader titration ranges or higher maximum doses of norepinephrine, our protocol restricted administration to a physiologic low-dose range below 0.05 µg/kg/min throughout surgery, consistent with the dose–response characteristics of norepinephrine under propofol–remifentanil anesthesia [9]. The infusion was discontinued after the anesthetic agents were stopped, reflecting its intended role in counteracting anesthesia-induced vasodilation [6,12]. Third, and most distinctively, unlike prior trials in which control patients were managed primarily with volume loading or alternative vasopressors rather than norepinephrine, our CONV group was managed with reactive norepinephrine at the attending anesthesiologist’s discretion—the same drug used preemptively in the CINE group—thereby directly testing whether a preemptive strategy provides incremental benefit over reactive management with the same vasopressor.

Consistent with this design, rescue medication was defined according to each group’s management strategy: any vasopressor use in the CONV group, versus dose escalation beyond 0.05 µg/kg/min or the addition of a second vasopressor in the CINE group. The lower rescue requirement in the CINE group (16.7% vs. 100%) therefore partly reflects this definitional difference inherent to the preemptive-versus-reactive comparison.

Regarding safety, we administered norepinephrine at a diluted concentration of 5 µg/mL through peripheral venous access in the upper arm. Although one previous perioperative recommendation suggested the use of an 18–20G catheter placed at or proximal to the antecubital fossa [20], our approach is supported by a pilot randomized trial demonstrating the feasibility and safety of low-concentration peripheral norepinephrine infusion through the forearm or arm [10] and is consistent with the subsequently published 2025 Association of Anaesthetists guideline [21], which recommends a 20–22G cannula placed in a large vein above the wrist and below the antecubital fossa for peripheral vasopressor administration. In addition, the preemptive low-dose norepinephrine infusion was generally no longer required after discontinuation of the anesthetic agents at the end of surgery, consistent with its primary role in counteracting anesthesia-induced hypotension.

This study has several limitations. First, attending anesthesiologists were aware of group allocation to allow appropriate real-time management of intraoperative hemodynamic instability, particularly with respect to rescue vasopressor administration in the control group. Although this may have introduced the clinician bias, we attempted to mitigate this risk by ensuring that the data analyst remained blinded to group assignment throughout the analysis. Second, tumor location differed between the groups. However, the closely related surgical position did not differ significantly between groups, and anesthesia duration and blood loss were comparable. These findings suggest that the intraoperative conditions most relevant to hemodynamic stability were similar between groups, despite the difference in tumor location. Furthermore, tumor location itself showed little or no association with the primary hemodynamic outcomes, indicating that its baseline imbalance was unlikely to have meaningfully influenced the between-group comparison. Third, the secondary outcomes, particularly rare postoperative complications, were not powered for statistical comparison; therefore the absence of significant differences should be interpreted as exploratory rather than as evidence of no effect. Fourth, the findings are most directly applicable to patients undergoing elective craniotomy for brain tumor resection under propofol–remifentanil TIVA at a single tertiary center, and whether the benefit extends to other surgical populations, anesthetic techniques, or practice settings remains to be established.

## 5. Conclusions

Preemptive low-dose norepinephrine infusion reduced the frequency and cumulative duration of moderate intraoperative hemodynamic instability during propofol–remifentanil TIVA for brain tumor resection, even when compared with a control strategy that allowed reactive norepinephrine administration. These findings suggest that incorporating preemptive physiologic-dose norepinephrine into standardized TIVA protocols may improve intraoperative hemodynamic control in neurosurgical patients and provide a rationale for adequately powered trials to determine whether this translates into improved clinical outcomes.

## Figures and Tables

**Table 1:** Baseline demographic and clinical characteristics.

Variable	CINE Group (*n* = 30)	CONV Group (*n* = 33)	*p*-Value	ASD
Age (years)	47 [37–59]	52 [41–61]	0.318	0.259
Sex (M/F)	19 (63.3%)	22 (66.7%)	0.782	0.070
Height (cm)	164.6 ± 8.7	162.7 ± 8.0	0.386	0.220
Weight (kg)	65 [58–75]	62 [54–65]	0.090	0.442
ASA physical status			0.778	
I	3 (10.0%)	5 (15.2%)		0.156
II	26 (86.7%)	26 (78.8%)		0.210
III	1 (3.3%)	2 (6.1%)		0.129
Hypertension	3 (10.0%)	8 (24.2%)	0.137	0.385
Diabetes mellitus	1 (3.3%)	2 (6.1%)	>0.999	0.129
COPD	1 (3.3%)	1 (3.0%)	>0.999	0.017
Current smoker	3 (10.0%)	2 (6.1%)	0.662	0.145
Beta-blocker	0 (0.0%)	1 (3.0%)	>0.999	0.250
Calcium channel blocker	2 (6.7%)	3 (9.1%)	>0.999	0.090
RAS inhibitor	2 (6.7%)	6 (18.2%)	0.261	0.355
Diuretics	0 (0.0%)	1 (3.0%)	>0.999	0.250
Preoperative hemoglobin (g/dL)	14.2 ± 1.5	13.6 ± 1.2	0.102	0.416
Tumor location			0.012	
Superficial supratentorial	15 (50.0%)	28 (84.8%)		0.749
Deep supratentorial	8 (26.7%)	3 (9.1%)		0.463
Infratentorial	7 (23.3%)	2 (6.1%)		0.494
Tumor type			0.705	
Meningioma	19 (63.6%)	25 (75.8%)		0.271
Glioma	8 (26.7%)	7 (21.2%)		0.128
Neuroma	1 (3.3%)	1 (3.0%)		0.017
Lymphoma	1 (3.3%)	0 (0.0%)		0.267
Epidermoid tumor	1 (3.3%)	0 (0.0%)		0.267

Values are presented as mean ± SD, median [IQR], or number of patients (proportion). ASD > 0.494 indicated imbalance. CINE, continuous infusion of norepinephrine; CONV, conventional; ASD, absolute standardized difference; ASA, American Society of Anesthesiologists; COPD, chronic obstructive pulmonary disease; RAS, renin–angiotensin system.

**Table 2:** Operative characteristics by group.

Variable	CINE Group (*n* = 30)	CONV Group (*n* = 33)	*p*-Value
Position			0.294
Supine	24 (80.0%)	29 (87.9%)	
Lateral ^a^	6 (20.0%)	3 (9.1%)	
Three quarter prone	0 (0.0%)	1 (3.0%)	
Duration of anesthesia (min)	439 ± 120	388 ± 104	0.072
Duration of surgery (min)	296 [262–387]	251 [213–339]	0.060
Fluid infused (mL/kg/h)	7.0 ± 2.1	7.6 ± 2.3	0.329
Urine output (mL/kg/h)	4.2 ± 1.5	4.8 ± 2.2	0.224
Blood loss (mL)	500 [400–700]	500 [350–500]	0.361
Anesthetic requirements			
Propofol (mg/kg/h)	6.7 [6.3–7.2]	6.5 [6.0–7.0]	0.504
Remifentanil (µg/kg/h)	6.8 [5.8–8.1]	6.5 [5.6–7.6]	0.237

Values are presented as mean ± SD, median [IQR] or number of patients (proportion). CINE, continuous infusion of norepinephrine; CONV, conventional. ^a^ Modified park bench position was included in the lateral category.

**Table 3:** Intraoperative hemodynamic instability and rescue medication use.

Variable	CINE Group (*n* = 30)	CONV Group (*n* = 33)	RR or Estimated Difference (95% CI)	*p*-Value
Moderate instability (MBP < 80% or >120% of baseline)				
No. of patients	30 (100%)	33 (100%)	N/A	N/A
Events per patient	6 [3–11]	9 [5–13]	−3.0 (−6.5 to 0)	0.045
Anesthesia time affected (%)	7.4 [3.6–12.4]	12.2 [6.8–21.4]	−4.84 (−9.59 to −0.02)	0.017
Severe instability (MBP < 70% or >130% of baseline)				
No. of patients	19 (63.3%)	24 (72.7%)	0.87 (0.62–1.23)	0.424
Events per patient	1 [0–2]	2 [0–3]	−1.0 (−2.0 to 1.0)	0.285
Anesthesia time affected (%)	1.2 [0.0–2.2]	1.8 [0.0–3.8]	−0.58 (−1.92 to 0.34)	0.127
Rescue medication required	5 (16.7%) ^a^	33 (100%) ^b^	0.17 (0.07–0.37)	<0.001
Ephedrine	1 (3.3%)	7 (21.2%)	0.16 (0.02–1.20)	0.056
Norepinephrine (NE)	5 (16.7%)	33 (100%)	0.17 (0.07–0.37)	<0.001

Values are presented as median [IQR] or number of patients (proportion). CINE, continuous infusion of norepinephrine; CONV, conventional; CI, confidence interval; MBP, mean blood pressure; NE, norepinephrine; RR, relative risk. ^a^ Ephedrine was administered to one patient; rescue norepinephrine was administered to all five patients. ^b^ Ephedrine was administered to seven patients; rescue norepinephrine was administered to all 33 patients.

**Table 4:** Perioperative changes in plasma lactate levels by group.

Plasma Lactate Level (mmol/L)	CINE Group (*n* = 30), Estimated Marginal Mean (95% CI) ^b^	CONV Group (*n* = 33), Estimated Marginal Mean (95% CI) ^b^	Mean Difference	*p*-Value ^c^
Intraoperative ^a^	1.50 (1.36–1.64)	1.37 (1.23–1.50)	0.13	0.169
End of operation	1.33 (1.21–1.46)	1.31 (1.19–1.43)	0.02	0.817

CINE, continuous infusion of norepinephrine; CI, confidence interval; CONV, conventional. ^a^ There were 10 missing data points in the CINE group and 7 in the CONV group for intraoperative lactate. ^b^ Estimated marginal mean values and 95% CIs based on a linear mixed-effects regression model, adjusted for baseline values. ^c^ Bonferroni-corrected *p*-value.

## Data Availability

The datasets generated during the current study are available from the corresponding author on reasonable request.

## Supplementary Materials

The following supporting information can be downloaded at https://www.mdpi.com/article/10.3390/jcm15135046/s1: Supplemental Table S1: Definitions of postoperative morbidity and mortality (within 30 days of surgery). Supplemental Table S2: Incidence of postoperative morbidity and mortality (within 30 days after surgery).
