# Supplementary material for: Preemptive Low-Dose Norepinephrine Infusion for Reducing Hemodynamic Instability During Craniotomy for Brain Tumor Resection Under Propofol–Remifentanil Total Intravenous Anesthesia: A Randomized Controlled Trial
Source: J Clin Med. 2026 Jun 29;15(13):5046. doi: 10.3390/jcm15135046 (PMC13362497; doi:10.3390/jcm15135046)
Supplement: Supplementary file 1 [file jcm-15-05046-s001.zip › jcm-4371720-supplementary.pdf]

Table S1. Definitions of postoperative morbidity and mortality (within 30 days of surgery)

Pleural effusion	Chest radiograph demonstrating blunting of the costophrenic angle, loss of sharp silhouette of the ipsilateral hemidiaphragm in upright position, displacement of adjacent anatomical structures, or (in supine position) a hazy opacity in one hemithorax with preserved vascular shadows
Atelectasis	Lung opacification with a shift of the mediastinum, hilum, or hemidiaphragm toward the affected area, accompanied by compensatory overinflation in the adjacent non-atelectatic lung
Respiratory infection	Patient has received antibiotics for a suspected respiratory infection and met one or more of the following criteria: new or changed sputum, new or changed lung opacities, fever, white blood cell count > 12×10^9 l^{-1}
Acute kidney injury	An increase in serum creatinine levels of ≥0.3 mg/dl within 48 h or an increase to ≥1.5 times the baseline within 7 days following surgery.
Myocardial infarction	Elevated serum cardiac biomarker values (preferably cardiac troponin) with at least one value above the 99th percentile upper reference limit, along with at least one of the following criteria: symptoms of ischaemia; new or presumed new significant ST-segment or T-wave changes on ECG, or new left bundle branch block; development of pathological Q waves on ECG; radiological or echocardiographic evidence of new loss of viable myocardium or new regional wall motion abnormality; and identification of an intracoronary thrombus at angiography or autopsy.
New cardiac arrhythmia	ECG evidence of atrial flutter, atrial fibrillation, or second- or third-degree atrioventricular conduction block.

Postoperative haemorrhage	Blood loss within 72 h after the start of surgery which would normally result in transfusion of blood
Stroke	An embolic, thrombotic or haemorrhagic cerebral event with persistent residual motor, sensory or cognitive dysfunction (e.g. hemiplegia, hemiparesis, aphasia, sensory deficit, impaired memory)
New neurological deficit	Any new postoperative neurological deficit not present preoperatively (motor, sensory, visual, cortical, or language), assessed by the neurosurgical team and considered attributable to surgical manipulation or resection rather than to a separately recorded postoperative hemorrhage or stroke.
Mortality	Death
Unexpected return to the operating room	An unexpected return to the operating room
Unexpected readmission to the NCU	Unexpected return to neurocritical care unit

ECG, electrocardiogram; NCU, neurocritical care unit.

Table S2. Incidence of postoperative morbidity and mortality (within 30 days after surgery)

**Outcome**	**CINE group (*n*=30)**	**Conventional group (*n*=33)**	**P-value**
Pulmonary			
Pleural effusion,	0	1 (3.0%)	>0.999
Atelectasis,	0	0	N/A
Pneumonia	0	0	N/A
Renal			
Acute kidney injury	0	0	N/A
Cardiac			
Myocardial infarction	0	0	N/A
New cardiac arrhythmia	0	0	N/A
Neurovascular			
Postoperative haemorrhage	1 (3.3%)	1 (3.0%)	>0.999
Stroke	1 (3.3%)	3 (9.1%)	0.614
New neurological deficit	6 (20.0%)	9 (27.3%)	0.498
Mortality	0	0	N/A
Unexpected return to the operating room	1 (3.3%)	2 (6.1%)	>0.999
Postoperative hospital stay (days)	9 [9–12]	9 [9–11]	0.705
Unexpected readmission to the NCU	0	1 (3.0%)	>0.999
Postoperative NCU stay (days)	1 [1–1]	1 [1–1]	0.581

Values are presented as median [IQR] or the number of patients (proportion). In the control group, one patient underwent external ventricular drain insertion due to central nervous system infection and hydrocephalus, whereas another patient underwent decompressive craniectomy for an epidural haematoma and was subsequently admitted to the NCU. In the CINE group, one patient underwent epidural haematoma removal but did not require NCU admission.

CINE, continuous infusion of norepinephrine; CI, confidence interval; NCU, neurocritical care unit; N/A, not applicable
